# Comparison of diagnostic sensitivity of [^18^F]fluoroestradiol and [^18^F]fluorodeoxyglucose positron emission tomography/computed tomography for breast cancer recurrence in patients with a history of estrogen receptor-positive primary breast cancer

**DOI:** 10.1186/s13550-020-00643-z

**Published:** 2020-05-24

**Authors:** Sun Young Chae, Hye Joo Son, Dong Yun Lee, Eonwoo Shin, Jungsu S. Oh, Seung Yeon Seo, Sora Baek, Ji Young Kim, Sae Jung Na, Dae Hyuk Moon

**Affiliations:** 1 Department of Nuclear Medicine, Asan Medical Center, University of Ulsan College of Medicine, Seoul, Republic of Korea; 2 Department of Nuclear Medicine, Kangdong Sacred Heart Hospital, Hallym University College of Medicine, Seoul, Republic of Korea; 3 Department of Nuclear Medicine, Guri Hospital of Hanyang University Medical Center, Hanyang University College of Medicine, Seoul, Republic of Korea; 4 Department of Radiology, Uijeongbu St. Mary’s Hospital, College of Medicine, The Catholic University of Korea, Seoul, Republic of Korea

**Keywords:** Breast cancer, Estrogen receptor, [^18^F]FES PET/CT, [^18^F]FDG PET/CT

## Abstract

**Background:**

To compare the diagnostic sensitivity of [^18^F]fluoroestradiol ([^18^F]FES) and [^18^F]fluorodeoxyglucose ([^18^F]FDG) positron emission tomography/computed tomography (PET/CT) for breast cancer recurrence in patients with estrogen receptor (ER)-positive primary breast cancer.

**Methods:**

Our database of consecutive patients enrolled in a previous prospective cohort study to assess [^18^F]FES PET/CT was reviewed to identify eligible patients who had ER-positive primary breast cancer with suspected first recurrence at presentation and who underwent [^18^F]FDG PET/CT. The sensitivity of qualitative [^18^F]FES and [^18^F]FDG PET/CT interpretations was assessed, comparing them with histological diagnoses.

**Results:**

Of the 46 enrolled patients, 45 were confirmed as having recurrent breast cancer, while one was diagnosed with chronic granulomatous inflammation. Forty (89%) patients were ER-positive, four (9%) were ER-negative, and one (2%) patient did not undergo an ER assay. The sensitivity of [^18^F]FES PET/CT was 71.1% (32/45, 95% CI, 55.7–83.6), while that of [^18^F]FDG PET/CT was 80.0% (36/45, 95% CI, 65.4–90.4) with a threshold of positive interpretation, and 93.3% (42/45, 95% CI, 81.7–98.6) when a threshold of equivocal was used. There was no significant difference in sensitivity between [^18^F]FES and [^18^F]FDG PET/CT (*P* = 0.48) with a threshold of positive [^18^F]FDG uptake, but the sensitivity of [^18^F]FDG was significantly higher than [^18^F]FES (*P* = 0.013) with a threshold of equivocal [^18^F]FDG uptake. One patient with a benign lesion showed negative [^18^F]FES but positive [^18^F]FDG uptake.

**Conclusions:**

The restaging of patients who had ER-positive primary breast cancer and present with recurrent disease may include [^18^F]FES PET/CT as an initial test when standard imaging studies are equivocal or suspicious.

## Background

Breast cancer is one of the most frequently diagnosed cancers and is the leading cause of cancer death in women [1]. About 75% of all breast cancer expresses estrogen receptor (ER) at the time of initial diagnosis [2]. The 20-year risk of distant recurrence after adjuvant endocrine therapy for ER-positive breast cancer ranges from 10 to 40% [3]. The diagnosis of recurrence and restaging are important for determining appropriate treatment [4].

Current guidelines on breast cancer recommend that the staging evaluation of women who present with recurrent breast cancer includes standard imaging studies [5, 6]. Positron emission tomography/computed tomography (PET/CT) with [^18^F]fluorodeoxyglucose ([^18^F]FDG) would only be used in situations where standard staging studies are equivocal or suspicious [5, 6]. Although clinical studies have indicated high diagnostic accuracy with sensitivities ranging from 81 to 100% and specificities ranging from 52 to 91% and have suggested the superiority of [^18^F]FDG PET/CT over standard imaging studies [7–11], these studies are largely retrospective with heterogeneous cohorts, and there are also methodological issues of concern [7, 10, 11]. The diagnostic accuracy was not separately determined in patients who presented with equivocal or suspicious imaging studies [8, 12–14]. In addition, both false-negative and false-positive [^18^F]FDG PET/CT results [7, 10] are inherent in [^18^F]FDG PET/CT [15, 16]. The consensus is that biopsy is more likely to provide useful information [6]. Moreover, the reference standard used in previous studies has ranged from histopathological diagnosis to clinical or radiological follow-up, and misclassification of disease might have led to positive or negative biases. Finally, the positivity threshold of [^18^F]FDG PET/CT may differ between studies, depending on how to determine the foci of increased [^18^F]FDG uptake are related to benign conditions. Equivocal decisions are therefore likely to be inevitable [8, 9, 11, 12, 17–19].

The histological and biological characteristics of breast cancer have an important impact on tumor visualization with [^18^F]FDG PET/CT [20]. [^18^F]FDG uptake correlates with histologic grade and tumor proliferation index [15, 21, 22], and [^18^F]FDG uptake is higher in ER-negative tumors [15]. Accordingly, the relatively lower [^18^F]FDG uptake in ER-positive breast cancer may affect the diagnostic accuracy. To date, the accuracy of [^18^F]FDG PET/CT for the diagnosis of recurrent breast cancer has not been separately reported in patients with ER-positive primary breast cancer.

PET/CT with [^18^F]fluoroestradiol ([^18^F]FES) provides a unique method to noninvasively assess molecular information on ER expression [23, 24]. The diagnostic sensitivity of [^18^F]FES PET/CT for the assessment of ER status in recurrent or metastatic lesions is good overall [25–31], which indicates a high sensitivity for diagnosing recurrent breast cancer in patients with a history of ER-positive primary breast cancer. Of particular importance is the very high specificity of [^18^F]FES PET/CT [25–31], with no false-positives having been reported in qualitative [^18^F]FES studies. Therefore, [^18^F]FES PET/CT can be used as an alternative to tissue biopsy and ER assays [31] and may improve clinical decision-making when conventional work-up is inconclusive [32]. To our knowledge, the use of [^18^F]FES PET/CT in the diagnosis of recurrent breast cancer in comparison with [^18^F]FDG PET/CT has not so far been studied.

Recently, we conducted a prospective cohort study to assess the diagnostic accuracy of [^18^F]FES PET/CT for ER status in recurrent lesions in patients with breast cancer [31]. The included participants were a consecutive series of all patients presenting with symptoms and imaging findings of recurrent breast cancer with histological confirmation of recurrent lesions. Most participants underwent [^18^F]FDG PET/CT for clinical reasons. The direct comparison of [^18^F]FDG and [^18^F]FES PET/CT in the same population is robust, as differences in potential modifiers associated with the population, study methods, and technologies can be eliminated. In the current study, we aimed to compare the diagnostic sensitivity of [^18^F]FDG and [^18^F]FES PET/CT for breast cancer recurrence in patients with ER-positive primary breast cancer who were enrolled in a previous cohort study.

## Materials and methods

### Study design

The study patients were identified from our database of a prospective patient cohort who underwent [^18^F]FES PET/CT for the assessment of ER status because of recurrent or metastatic breast cancer lesions [31]. The patients’ medical histories and [^18^F]FDG PET/CT studies performed to identify unsuspected regional nodal disease and/or distant metastases were retrospectively reviewed. Our institutional review board approved this study protocol and waived the requirement for informed consent. This study was conducted in accordance with the Declaration of Helsinki and our institutional guidelines.

### Patients

All eligible patients had ER-positive primary breast cancer with suspected first recurrence at presentation, were enrolled in a previous prospective cohort study to assess [^18^F]FES PET/CT, and underwent [^18^F]FDG PET/CT within 60 days before needle biopsy or surgery. Patients were excluded if they had ER-negative or ER-unknown primary breast cancer. The complete selection criteria, setting, location, and dates of the prospective cohort study were previously reported [31]. The patient population used in this study represented a consecutive sample of eligible patients.

### [^18^F]FES and [^18^F]FDG PET/CT procedure

The production of [^18^F]FES was described previously [33]. PET/CT imaging was performed from the skull base to upper thigh using a Discovery PET/CT 690 or 710 scanner (GE Healthcare), 80–100 min after intravenous injection of 111–222 MBq (3–6 mCi) of [^18^F]FES [31]. PET/CT images were reconstructed using the manufacturer-provided iterative algorithm with 4 iterations and 18 subsets.

[^18^F]FDG PET/CT images were obtained from the skull base-to-upper thigh using one of several different PET/CT scanners (Biograph Sensation 16 or Biograph TruePoint 40, Siemens Healthineers; or Discovery PET/CT 690, 690 Elite, or 710, GE Healthcare), 50–70 min after intravenous injection of 5.2–7.4 MBq/kg (0.14–0.2 mCi/kg) of [^18^F]FDG as described in a previous report [34].

### Histological assessment

Experienced breast pathologists who were blinded to the results of the [^18^F]FES PET/CT reviewed core-needle biopsy and surgical samples in accordance with routine standard procedures. The recurrent or metastatic specimens taken from the breast, liver, ovary, uterus, or bone were excluded from the analysis. Clinical information and other laboratory test results were available to the assessors of the histological examinations. Immunostaining for ER was performed using the 6F11 mouse monoclonal antibody (NCL-ER-6F11, Novocastra Laboratories), as previously described [35]. Semi-quantitative ER receptor expression was evaluated according to the Allred score [36]. ER status was considered positive if Allred scores were 3 or higher.

### Analysis of PET/CT imaging

The recurrent lesion from which a sample was taken for histological examination was selected as the reference lesion for the sensitivity analysis, as reported previously [31]. Three board-certified nuclear medicine physicians (S.B., J.Y.K., and S.J.N.) who were blinded to all patient information including histological diagnosis, independently assessed the [^18^F]FES PET/CT images in a random sequence. The physicians qualitatively assessed the [^18^F]FES uptake of reference lesions, as well as up to five additional non-reference lesions in the order of highest intensity relative to background activity [25, 37]. Lesions were interpreted as ER-positive breast cancer when the majority of [^18^F]FES assessments were positive.

Two board-certified nuclear medicine physicians (H.J.S. and D.Y.L.) interpreted the [^18^F]FDG PET/CT images in consensus using a syngo.via image display system (VB20A, Siemens Healthineers), with the images displayed in a random sequence. These two physicians were also blinded to all patient information and the [^18^F]FES PET/CT images; they only had knowledge of the location of the biopsy sites for [^18^F]FDG PET/CT image evaluation. The lesions were classified as positive, equivocal, or negative lesions by qualitative analysis. An [^18^F]FDG focus was interpreted as negative when it was related to a non-malignant process or the physiological biodistribution of [^18^F]FDG. Focally abnormal [^18^F]FDG uptake higher than that of surrounding tissues and that could not be related to non-malignant or physiological uptake was interpreted as positive. [^18^F]FDG uptake that could not be characterized was defined as equivocal [17].

For quantitative analysis, the readers calculated the maximal standardized uptake value (SUV) as SUV = activity (Bq/g)/[injected activity (Bq)/body weight (g)]. The [^18^F]FDG SUV results were harmonized across different PET/CT scanners by matching the recovery coefficient profiles without partial volume correction, as described previously [38].

### Statistical analysis

All data are presented as median values with interquartile range (IQR) for continuous variables, and numbers (%) for categorical variables. A two-sided *P* value of less than 0.05 was considered as the threshold for significance. Quantitative parameters were compared using the Mann-Whitney *U* test or Kruskal-Wallis *H* test and categorical data were compared using the McNemar test. All statistical tests were conducted using SPSS Statistics for Windows (version 21, IBM Company). The sensitivity of PET/CT was defined as the probability that a PET/CT result would show positive uptake when the patient had histologically proven recurrent breast cancer. Estimates are presented with 95% Clopper-Pearson exact confidence intervals (CI).

## Results

### Patients

**Fig. 1:**
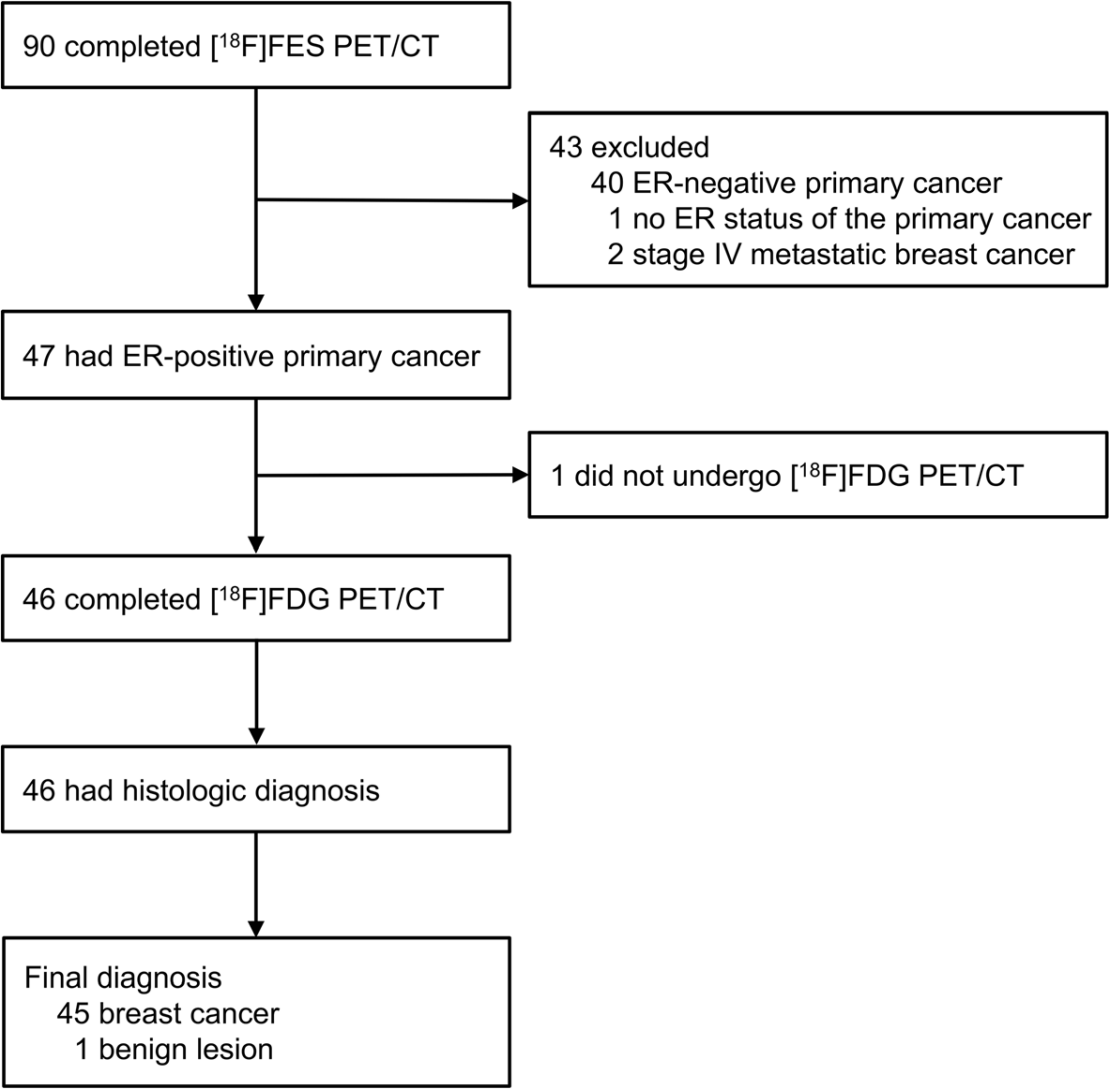
Flow of patients

Between November 2013 and November 2016, 90 patients completed [^18^F]FES PET/CT [31]. Figure 1 shows the flow of patients, with the patients who were excluded from this study being noted. Of 47 women with ER-positive primary cancer, 46 who underwent both [^18^F]FES and [^18^F]FDG PET/CT were included in this study. Thirty-five of 46 patients (76%) underwent [^18^F]FDG PET/CT before needle biopsy or surgery, 22 of whom were initially diagnosed as having recurrent tumors by ultrasonography or computed tomography, and the 13 of whom were detected by [^18^F]FDG PET/CT. The remaining 11 (24%) had [^18^F]FDG PET/CT on the day of or after tissue biopsy. The clinical and histological characteristics are listed in Table 1. Forty-five patients were confirmed as having breast cancer, but one patient was diagnosed with chronic granulomatous inflammation. Of the 44 patients with recurrent breast cancer who underwent an immunohistochemical study, four (9%) were ER-negative. One patient had a fine-needle aspiration biopsy that did not provide enough cells for immunohistochemical study.

**Table 1:** Baseline demographic, clinical, and histological characteristics

Characteristic	Patients (*n* = 46)
Age, years	56 (49–61)
Menopausal status	
Premenopausal	13 (28%)
Postmenopausal	33 (72%)
Body mass index, kg/m^2^	22.7 (20.5–25.1)
Time interval from surgery to recurrence, months	94 (56–130)
Pathology of the primary carcinoma	
Invasive ductal carcinoma	44 (96%)
Invasive lobular carcinoma	2 (4%)
Pathological diagnosis of suspected recurrence or metastatic lesions	
Regional lymph node*^†^	19 (41%)
Distant lymph node^†^	10 (22%)
Lung^†^	9 (20%)
Chest wall^†^	6 (13%)
Pleura^†^	1 (2%)
Chronic granulomatous inflammation	1 (2%)
ER Allred score^‡^	
0–2	4 (9%)
3–6	9 (20%)
7–8	31 (71%)
Progesterone receptor^‡^	
Positive	18 (41%)
Negative	23 (52%)
Not assessed	3 (7%)
HER2^‡^	
Positive	5 (11%)
Negative	34 (77%)
Not assessed	5 (11%)

Data are median (IQR) or *n* (%)

*Metastases in ipsilateral axillary, internal mammary, supraclavicular, or infraclavicular lymph node(s)

^†^Histologically confirmed invasive breast cancer

^‡^The number of patients who underwent an immunohistochemical assay for a recurrent lesion = 44

### Sensitivity of [^18^F]FES and [^18^F]FDG PET/CT

The median time between PET/CT and biopsy or surgery was 5 days (IQR 1–11) for [^18^F]FES and 11 days (IQR 5–21) for [^18^F]FDG. The median time interval between [^18^F]FDG and [^18^F]FES PET/CT was 10 days (IQR 7–19). All but three patients underwent the [^18^F]FDG PET/CT at our institution. The injected dose of [^18^F]FDG ranged from 218 to 440 MBq (5.9–11.9 mCi). The uptake time before [^18^F]FDG PET/CT ranged from 50 to 67 min. The median blood glucose level was 99 mg/dl (IQR 88–108).

Table 2 shows the results of the qualitative interpretations of [^18^F]FES and [^18^F]FDG uptake in the 45 patients with recurrent breast cancer. Of these 45 patients, [^18^F]FES PET/CT was positive in 32, and the sensitivity for diagnosing recurrent breast cancer was 71.1% (95% CI, 55.7–83.6). [^18^F]FDG was positive in 36 when a positive interpretation was defined as the threshold, and 42 when a positive or equivocal interpretation was used, with the sensitivities being 80.0% (95% CI, 65.4–90.4) and 93.3% (95% CI, 81.7–98.6), respectively. When a threshold of positive [^18^F]FDG uptake was used, there was no significant difference in sensitivity between [^18^F]FES and [^18^F]FDG PET/CT (*P* = 0.48). However, when a threshold of equivocal uptake was used, the sensitivity of [^18^F]FDG PET/CT was significantly higher than that of [^18^F]FES PET/CT (*P* = 0.013).

**Table 2:** Qualitative interpretation of [^18^F]FES and [^18^F]FDG PET/CT

	[^18^F]FES PET/CT	[^18^F]FDG PET/CT			Total
		Positive	Equivocal	Negative	
All recurrent breast cancer	Positive	25	5	2	32
	Negative	11 (4*)	1	1	13
	Total	36	6	3	45
ER-positive recurrent breast cancer	Positive	24	5	2	31
	Negative	7	1	1	9
	Total	31	6	3	40

*Number of patients with ER-negative recurrent breast cancer

**Fig. 2:**
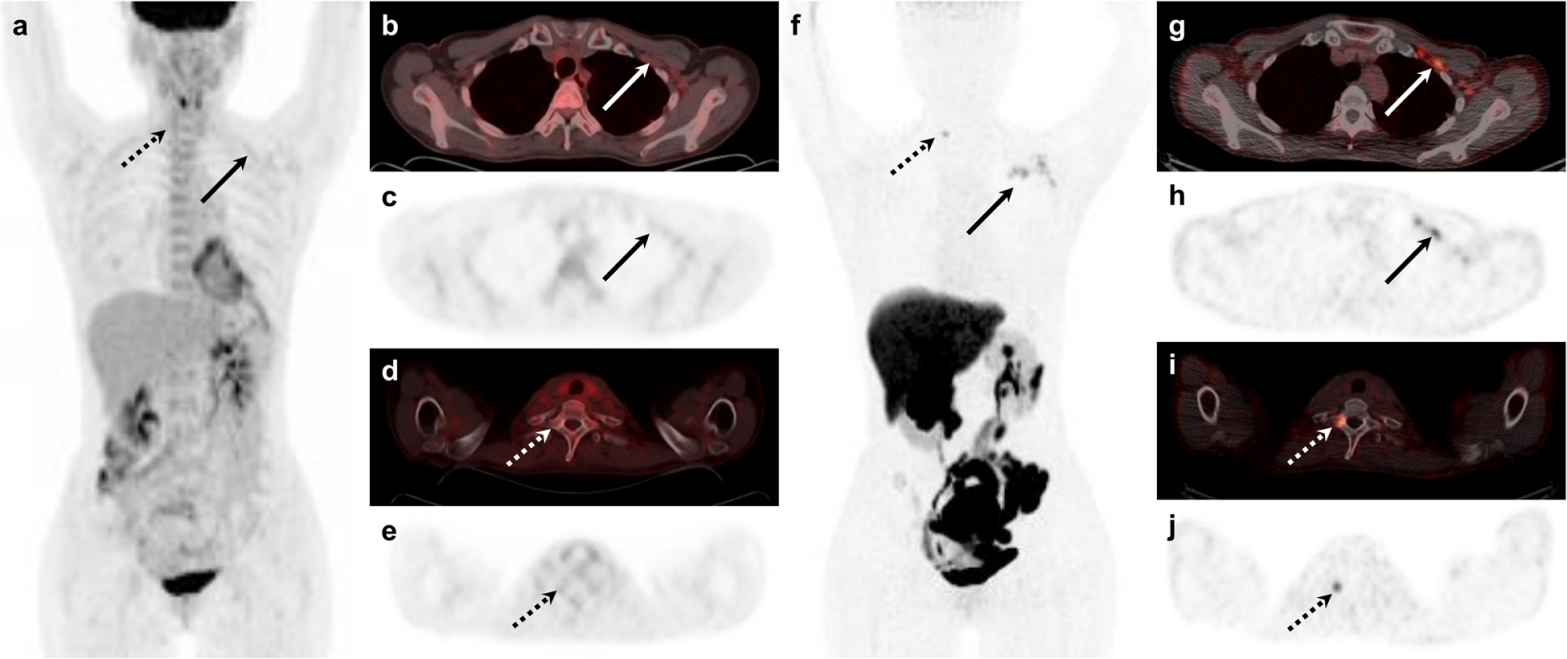
[^18^F]FDG and [^18^F]FES PET/CT images of a 46-year-old female with lobular carcinoma and histologically confirmed ER-positive recurrence in the left axillary level II lymph node (Allred score = 8). Maximum intensity projection (**a**) and transaxial [^18^F]FDG images (**b**–**e**) show equivocal uptake in the left axillary lymph node (arrows) and physiologic bone uptake in the thoracic spine (dotted arrows). However, positive [^18^F]FES uptake is seen in the axillary lymph nodes (**f**–**h**; arrows) and the first thoracic vertebra (**f**, **i**, **j**; dotted arrows)

Of the 40 patients with ER-positive recurrent breast cancer, [^18^F]FES PET/CT was positive in 31 and [^18^F]FDG PET/CT with thresholds of positive or equivocal uptake was positive in 31 and 37 patients, resulting in sensitivities of 77.5% (95% CI, 61.5–89.2), 77.5% (95% CI, 61.5–89.2), and 92.5% (95% CI, 79.6–98.4), respectively, with there being no significant difference between them (*P* > 0.10). Five of six patients with equivocal [^18^F]FDG and two of three with negative [^18^F]FDG uptake showed a positive [^18^F]FES PET/CT result (Table 2). One patient with ER-positive recurrence from primary invasive lobular carcinoma was graded as equivocal on [^18^F]FDG PET/CT but positive on [^18^F]FES PET/CT (Fig. 2). The other patient with ER-positive invasive ductal primary cancer had a biopsy-confirmed benign lesion that showed negative [^18^F]FES but positive [^18^F]FDG uptake.

The maximum SUV of [^18^F]FES differed significantly between the positive (median, 5.8; range, 1.8–17.1) and negative (median, 1.3; range, 0.8–2.1) qualitative interpretations (*P <* 0.0001). There was also a significant difference in the maximum SUVs of [^18^F]FDG between positive (median, 4.9; range, 1.4–13.7), equivocal (median, 2.6; range, 1.6–3.3), and negative (median, 0.8; range, 0.6–1.0) interpretations (*P* = 0.0012).

### Assessment of whole-body tumor burden by [^18^F]FES PET/CT

**Fig. 3:**
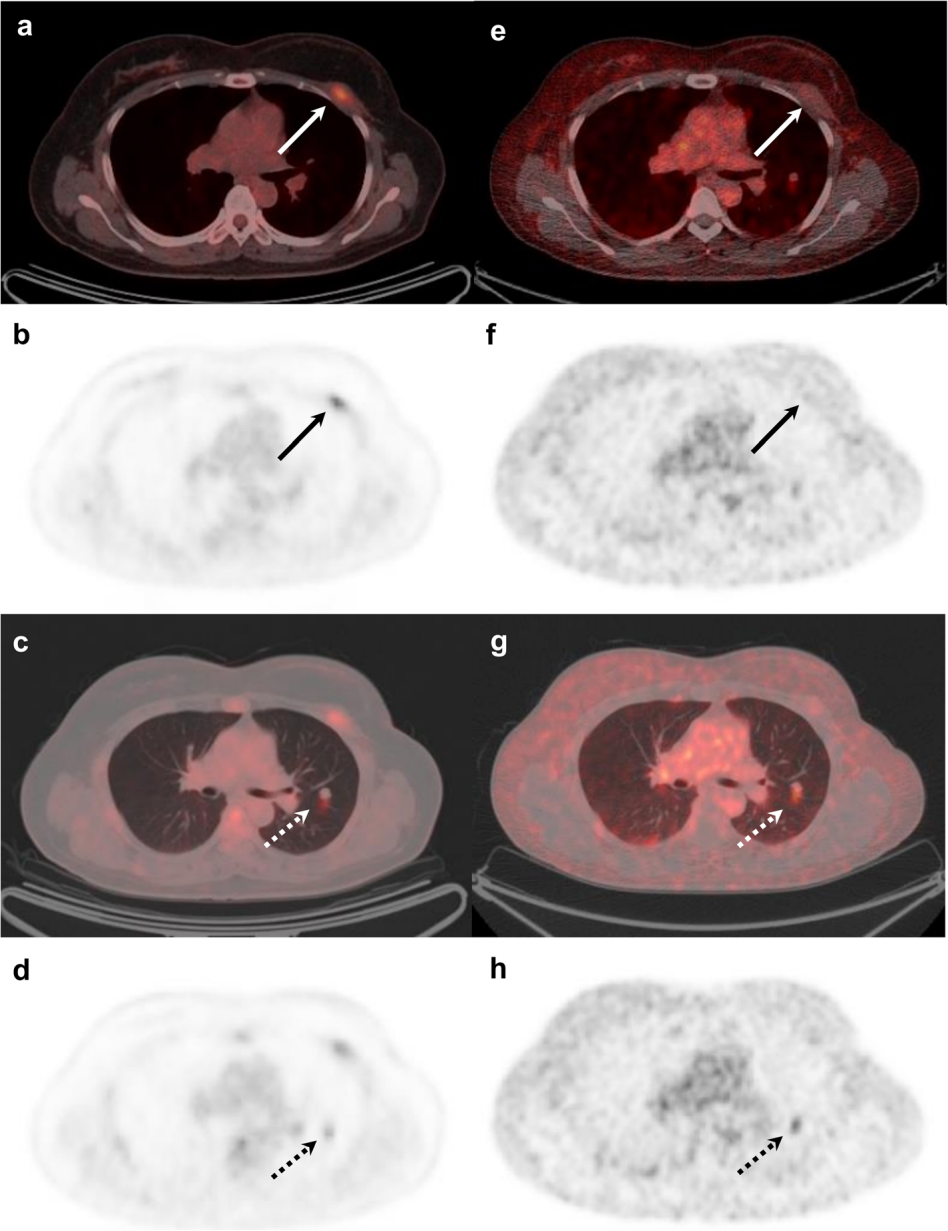
[^18^F]FDG and [^18^F]FES PET/CT images of a 51-year-old female with histologically confirmed ER-positive recurrence (Allred score 5) in the chest wall. Transaxial [^18^F]FDG images show positive uptake in the left chest wall recurrence (**a**, **b**; arrows). Additional positive [^18^F]FDG uptake is seen in the left lung (**c**, **d**; dotted arrows). By contrast, [^18^F]FES PET/CT is negative in the chest wall (**e**, **f**; arrows) and positive only in the left lung (**g**, **h**; dotted arrows). The left lung lesion was later confirmed as ER-positive metastatic breast cancer

Of the 45 patients with recurrent breast cancer, 25 patients (56%) had a total of 47 [^18^F]FES-positive non-reference lesions (Fig. 2). All these patients had ER-positive reference lesions. These [^18^F]FES-positive lesions were located in the bone (*n* = 10), regional lymph nodes (*n* = 10), distant lymph nodes (*n* = 11), lung (*n* = 11), pleura (*n* = 4), and breast (*n* = 1). Interestingly, three of nine patients who had ER-positive/[^18^F]FES-negative reference lesions had [^18^F]FES-positive non-reference lesions. One non-reference lesion of the three patients was confirmed as ER-positive metastatic breast cancer by histologic analysis (Fig. 3). If these three patients were to be added to the calculation of the sensitivity of [^18^F]FES, the figure would be 77.8% (35/45, 95% CI, 62.9–88.8).

## Discussion

In this study, we showed that [^18^F]FES PET/CT had comparable diagnostic sensitivity to [^18^F]FDG PET/CT for the diagnosis of recurrent breast cancer when positive [^18^F]FDG uptake was used as a threshold. However, [^18^F]FES PET/CT had significantly lower sensitivity than [^18^F]FDG PET/CT if equivocal [^18^F]FDG uptake was included as a positive threshold. When ER-positive recurrent breast cancer was analyzed, there was no significant difference in sensitivity between [^18^F]FDG and [^18^F]FES PET/CT. One patient with a benign lesion showed true-negative [^18^F]FES but false-positive [^18^F]FDG uptake. To the best of our knowledge, this is the first study evaluating the diagnostic performance of [^18^F]FES and [^18^F]FDG PET/CT relative to histological results in patients with ER-positive primary breast cancer.

In this study, we included only ER-positive primary cancer patients in whom [^18^F]FES PET/CT would be likely to show high sensitivity. The sensitivity of [^18^F]FES PET/CT was comparable to or lower than that of [^18^F]FDG, depending on the threshold used. All four patients with ER-negative recurrence showed negative [^18^F]FES PET/CT. However, the false-negative [^18^F]FES PET/CT results were of relevance, because discordant ER expression between primary and recurrent breast cancer was accurately determined. We also had nine false-negative patients who displayed ER-positive immunohistochemistry but negative [^18^F]FES. As seven of the nine [^18^F]FES-negative patients showed a positive [^18^F]FDG uptake, the false-negative results cannot be explained by the limited spatial resolution of PET/CT, but rather reflect the low ER expression levels in tumor, or inherent differences between ER assays and [^18^F]FES uptake [31, 39]. ER-positive but [^18^F]FES-negative tumors may represent a functionally endocrine therapy-resistant breast cancer [23, 24]. Further studies are required to clearly characterize those patients with recurrent breast cancer who are ER-positive but [^18^F]FES-negative.

An [^18^F]FES PET/CT strategy for diagnosing recurrent breast cancer would have false-negative results, which would generally lead to delayed treatment [7]. Therefore, [^18^F]FDG PET/CT may be offered for patients who are [^18^F]FES-negative. Despite the theoretically lower sensitivity of [^18^F]FES PET/CT for breast cancer recurrence, positive [^18^F]FES uptake could replace histological diagnosis of recurrent breast cancer and positive ER expression [31] and could lead to a reduction in the number of false-positive diagnoses and biopsies compared with [^18^F]FDG PET/CT [40]. [^18^F]FES PET/CT would be of additional value in whole-body imaging for tumors expressing ER, to identify the subset of patients with mixed [^18^F]FES uptake, as shown in the three patients with heterogeneous uptake [41]. However, an [^18^F]FDG PET/CT-first strategy may have lower false-negative results. Nevertheless, positive [^18^F]FDG results still require histological confirmation and assessment of ER status, and even negative [^18^F]FDG cannot completely exclude the possibility of recurrent breast cancer; therefore, biopsies of possible breast cancer recurrence may still be performed. If collecting a biopsy sample is not feasible or associated with risk, positive [^18^F]FES PET/CT results can adequately diagnose ER-positive recurrent breast cancer and represent an alternative to histological assessment. Our results suggest that although [^18^F]FES PET/CT may have a slightly lower sensitivity than [^18^F]FDG, staging evaluation of women who had ER-positive primary breast cancer and then present with recurrent disease could include [^18^F]FES PET/CT. An [^18^F]FES PET/CT-first strategy for recurrent cancer may lead to changes in patient management and have an impact on outcomes.

Diagnostic accuracy studies are at risk of bias due to shortcomings in their design and conduct, and the results may not be applicable to other patient populations [42]. We retrospectively reviewed our database of a prospective cohort who underwent [^18^F]FES PET/CT for the assessment of ER status in recurrent lesions, and included consecutive patients who were typical of those presenting for first breast cancer recurrence. However, our patient inclusion and exclusion criteria may have resulted in potential risks and limited applicability. We enrolled only those patients who had histological confirmation and who underwent [^18^F]FES PET/CT for restaging to guide optimal therapy. The diagnostic sensitivity may be higher if lesions are large enough for a tissue biopsy to be attained. In addition, PET/CT studies might have not been performed in situations where standard staging studies are equivocal or suspicious, as recommended by the guidelines. Nevertheless, we believe that the primary purpose of this study, which was to compare sensitivity between [^18^F]FDG and [^18^F]FES PET/CT, is unlikely to be influenced by our patient selection. We excluded only one patient who had no [^18^F]FDG PET/CT. In most patients, the [^18^F]FDG studies were performed for clinical reasons. There are no other potential risks of bias or applicability regarding the quality of the diagnostic sensitivity data [43]. Furthermore, the qualitative [^18^F]FES positivity threshold could be precisely determined, as suggested by a very low *P* level for the differences in SUV results between the dichotomous qualitative [^18^F]FES categories.

There are several limitations to this study. First, we enrolled only one patient with a benign inflammatory lesion. It was not possible to directly demonstrate a high specificity of [^18^F]FES PET/CT compared with [^18^F]FDG PET/CT; however, the main concern was how the sensitivity of [^18^F]FES differs from that of [^18^F]FDG in patients with ER-positive primary breast cancer. A comparison of the specificity of [^18^F]FES and [^18^F]FDG PET/CT can be inferred from previous studies. Second, we did not include asymptomatic patients with elevated tumor markers. Our results may not be applicable to the specific setting of rising tumor markers. Third, this study may have verification bias in which the positive results of [^18^F]FDG PET/CT in 13 patients could affect the biopsy decision leading to a high sensitivity estimate. Nonetheless, the sensitivity of [^18^F]FES PET/CT was comparable to that of [^18^F]FDG PET/CT when positive interpretation was defined as positive for the presence of recurrent breast cancer. Finally, we did not assess the diagnostic value of [^18^F]FDG and [^18^F]FES PET/CT in addition to or instead of standard imaging tests. [^18^F]FES PET/CT can be performed as an initial test in the setting of recurrent disease when standard imaging studies are equivocal or suspicious.

## Conclusion

The sensitivity of [^18^F]FES PET/CT in patients who had ER-positive breast cancer and presented with recurrent disease was comparable to or lower than that of ^18^F-FDG, depending on the threshold used. Although we were not able to directly demonstrate a high specificity in this study, the well-established high specificity indicates that positive [^18^F]FES PET/CT results can adequately diagnose ER-positive recurrent breast cancer. [^18^F]FES PET/CT would be of additional value in whole-body imaging of tumor ER expression, to identify additional patients with recurrence with mixed [^18^F]FES uptake. We conclude that the restaging of women who have ER-positive primary breast cancer and present with recurrent disease may include [^18^F]FES PET/CT. Large-scale prospective intervention studies are needed to investigate the potential utility of [^18^F]FES PET/CT as a replacement for, rather than an addition to, standard imaging studies, and to determine the impact of [^18^F]FES PET/CT on patient outcomes.

## Data Availability

The datasets generated and/or analyzed during the current study are available from the corresponding author on reasonable request.

## Abbreviations

[18F]FES: [18F]fluoroestradiol
[18F]FDG: [18F]fluorodoxyglucose
PET/CT: Positron emission tomography/computed tomography
ER: Estrogen receptor
IQR: Interquartile range
CI: Confidence intervals
